# How Do Men and Women Perceive a High-Stakes Test Situation?

**DOI:** 10.3389/fpsyg.2018.02216

**Published:** 2018-12-04

**Authors:** Julia E. M. Leiner, Thomas Scherndl, Tuulia M. Ortner

**Affiliations:** Department of Psychology University of Salzburg, Salzburg, Austria

**Keywords:** test situation perception, test anxiety, sex differences, fairness perception, test performance

## Abstract

The results of some high-stakes aptitude tests in Austria have revealed sex differences. We suggest that such discrepancies are mediated not principally by differences in aptitudes, skills, and knowledge but sex differences in test takers' perceptions of the test situation. Furthermore, previous research has indicated that candidates' evaluations of the fairness of the testing tool are of great importance from an institutional point of view because such perceptions are known to influence an organization's attractiveness. In this study, we aimed to investigate how women and men perceive and evaluate certain aspects of a high-stakes test situation by using the results and evaluations of an actual medical school aptitude test (747 applicants; 59% women). Test takers voluntarily evaluated the test situation and rated specific aspects of it (e.g., the fairness of the selection tool) and provided information regarding their test anxiety immediately after they completed the 4-h test. Data analyses indicated small, albeit significant sex differences in participants' perceptions of the test. Men described the test situation as slightly giving more opportunity to socialize and possessing more opportunity to deceive than women did. Furthermore, the perception of the test situation did not directly predict the test results, but it served as a moderator for the indirect effect of test anxiety on test results. By contrast, there were significant direct effects but no indirect effects of situation perception on evaluations of the fairness of the selection tool: The more the test situation was perceived as a high-pressure situation, the lower the fairness ratings of the testing tool. Results were discussed with reference to gender roles and test fairness.

## Introduction

Imagine an important assessment situation, for example, a high-stakes test situation with 100 test takers. When viewed only superficially, there is just one situation, which appears to be very much the same for every test taker. However, there may be up to 100 different impressions because each individual may perceive the same situation in a different way. In this study, we aim to address the issue of differences in perceptions of a competitive standardized high-stakes test situation. The focus lies on sex-related differences in people's perceptions of the situation and the possible effects of these perceptions on test performance. The goal is to initiate a new approach for assessing sex differences in competitive environments.

In many competitive areas, for example, in academic science, professional and managerial senior positions, and assessments, women tend to be outperformed by men. In the European Union, women are underrepresented in senior academic positions (EU, 2012), and larger numbers of board members in European and U.S. companies are represented by men (Backus et al., 2016). Although women and men do not differ considerably in their skills and abilities (see Hyde et al., 1990), aptitude tests have painted a different picture with respect to test performance (see Mau and Lynn, 2001): Analyses have revealed cross-national sex differences in performances on college and aptitude tests (Else-Quest et al., 2010; Salchegger and Suchan, 2018). Whereas in general differences in verbal ability and writing tests favor girls (Reilly et al., 2018), differences in math tests favor boys (Reilly et al., 2015). These differences also apply to high-stakes tests, such as the Graduate Record Exam in the U.S. (see e.g., https://www.prepscholar.com/gre/blog/average-gre-scores/). However, with reference to cognitive performance, research has revealed that sex differences that favor male test takers tend to occur particularly in competitive situations, indicated by an increase in the performance of men and basically no change in the performance of women, even when women's performances are similar to men's in non-competitive environments (Gneezy et al., 2003; Niederle and Vesterlund, 2007, 2010). In Austria, test scores on public medical high-stakes aptitudes tests have been under public scrutiny for years because of sex differences in test scores (Pfarrhofer, 2017). Although a larger percentage of women (60%) compared with men (40%) took the test in 2017, women represented only 53% of the test takers who were accepted to a university, thus indicating that they scored lower than men (see Pfarrhofer, 2017). Because the relevance of test scores for decisions in the educational system has increased in Europe in recent years (e.g., see the growing number of subjects with entrance exams at Cambridge University and Oxford University; Turner et al., 2017; or the establishment of new entrance tests at German universities after a decision made by the German Constitutional Court in 2017, see Konegen-Grenier, 2018), attention has also been directed toward the topic of test bias and fairness (e.g., Kaufmann, 2010; Fischer et al., 2013; Aguinis et al., 2016). If test scores on group levels are systematically affected by factors that are not intended to be measured by the test, the test provides inaccurate and unfair scoring. According to Helms' (2006) quantitative model, differential performance between groups may stem from individuals' interpretations of test situations that are based on differential past experiences. Interpretations and experiences in test situations and their impact on women's and men's test scores have been insufficiently investigated so far. Therefore, the present study aimed to explore the perceptions of women and men in a high-stakes test situation.

### Systematic Measurement Error: Construct-Irrelevant Variance

When it comes to the assessment of achievement-related variables, the test design as well as the situational circumstances surrounding the assessment situation should allow test takers to show their maximal performance (e.g., Willingham and Cole, 1997). Codes of conduct and standards for test fairness (e.g., the Standards for Educational and Psychological Testing; American Educational Research, Association, American Psychological, Association, and National Council on Measurement in Education, 2014) state that the test situation should further aim to provide comparable opportunities for all test takers to apply the skills, abilities, and knowledge they possess. From a psychometric perspective, the part of the overall variability of the scores that can be attributed to construct-relevant variance should be maximized, whereas the influence of factors that are irrelevant to the construct should be minimized (Stone and Cook, 2016). With respect to measurement error, the literature has distinguished random error from systematic error (see, e.g., Cote and Buckley, 1987). Subsequently, systematic measurement errors are caused by factors that affect measurement outcomes systematically, resulting in a systematic decrease in test scores for an individual test taker or a group of test takers.

Haladyna and Downing (2004) presented a taxonomy for the study of systematic errors associated with construct-irrelevant variance threatening test score interpretation and addressed test anxiety as one of the most common sources. Test anxiety as a trait characteristic, defined as “the tendency to view with alarm the consequences of inadequate performance in an evaluative situation” (Sarason, 1978, p. 213) has been investigated for decades, with women reporting higher occurrences of test anxiety than men (Hembree, 1988; Zeidner, 1990). Research has revealed that test anxiety can impair those who are affected in different ways: Highly test-anxious people are more sensitive to environments that emphasize competition (Hancock, 2001) and tend to view test situations in particular as personally threatening (Sarason and Sarason, 1990). Test anxiety was found to be associated with academic self-concept (Zeidner and Schleyer, 1998) and was identified as affecting academic performance (Chapell et al., 2005). With respect to the underlying mechanism that causes performance to decrease, test anxiety was revealed to impair working memory capacity (Ashcraft and Kirk, 2001) because highly anxious individuals are believed to use more processing resources by worrying than individuals low on anxiety (Eysenck and Calvo, 1992). Furthermore, anxiety was found to lead individuals to show a more self-focusing strategy instead of a task-focusing tendency (Hancock, 2001). These mechanisms could serve as explanations for the underperformance of women on achievement tests.

Based on qualitative and quantitative data, Bonaccio and Reeve (2010) developed a framework of perceived sources of test anxiety: Besides students' perceptions of the test as well as their perceptions of themselves, the test-taking situation was revealed to be an important source of test anxiety. With respect to reactions to test situations, Steele (1997) was the first to introduce *stereotype threat* as a source of bias on standardized tests. Negative stereotypes were identified as a core characteristic of this phenomenon because self-threats were revealed to interfere with the targets' test performance. Experiments have shown, for example, that women performed worse than men when both groups were explicitly told that this test should show sex differences. In contrast, these differences in women's and men's test performance vanished when the same test was presented stereotype-free (see Spencer et al., 1999). Schmader and Johns (2003) reported that stereotype threat reduced cognitive capacity, which led to lower performance for the stereotyped group. Steele (1997) stressed performance differences caused by stereotype threat as an effect of the situation: Extra situational pressure sets up the frame for attributions of gender-related ability limitations. Research indicated that stereotype threat led to higher numbers of negative thoughts (Cadinu et al., 2005), whereas negative thoughts were identified as related to the cognitive component of test anxiety (Cassady and Johnson, 2002).

### Situation Perception

According to an early statement made by Lewin (1946), people and their environments are interwoven and cannot be separated or studied independently. Situations provide information that is distinctively processed by each individual (Sarason, 1978), thus influencing people's perceptions (e.g., how to encode the situation, expected outcomes, and their subjective value) and thereby affecting the way individuals think and act under such conditions (Mischel, 1977). Considering the interaction of persons and situations, Mischel (1977) shifted the focus to draw attention to the issue of “When are situations most likely to exert powerful effects […]?” (p. 346), thus addressing their potential influence on individual behavior. His claim refers to so-called *strong* situations, which provide clear incentives and normative expectations of behavior—criteria that are met in a test situation because of their high standardization and rules of conduct. At the other end are *weak* situations, which lack environmental cues for performance. Nevertheless, Cooper and Withey (2009) extended this theory by more recently postulating a *continuum* between strong situations (resulting in main effects of only the situation on behavior) and weak situations (resulting in main effects of only personality on behavior) by proposing that an individual's personality also affects perceptions and reactions in strong situations.

In his model, Rauthmann (2012) proposed that people's unique impressions lead to three components of variance in ratings of situations: perceiver variance, situation variance, and perceiver × situation variance (*Situation Perception Components Model*; Rauthmann, 2012). With reference to the terminology employed in current approaches in research on situations, *cues* are defined as objectively quantifiable stimuli that need to be processed by a perceptual system to be interpreted with reference to its content. Each situation is made up by several cues (see Rauthmann et al., 2015), which can be associated with psychological meanings (e.g., pleasant or negative); *characteristics* (also referred to as qualities or features) determine the psychological meaning of detected cues, embracing the situation's psychological “power” (Edwards and Templeton, 2005; Rauthmann et al., 2014). Situations containing similar cues and/or similar combinations of characteristics and sharing important aspects of their psychological meanings can be summarized as *classes* of situations. With reference to these different classes, current approaches aim to establish empirically based “class taxonomies” as a system of categories that integrates all possible situations. Recently, analyses of a large and multinational set of data from a questionnaire for assessing situational characteristics (Situational Q-Sort; Wagerman and Funder, 2009) led to a model represented by a structure of eight psychological characteristics relevant for describing situations (Rauthmann et al., 2014): The widely recognized Situational Eight DIAMONDS model (e.g., Rauthmann and Sherman, 2016, 2017; Horstmann and Ziegler, 2018; Rauthmann et al., 2018) comprises the following dimensions with original sample questions (see Rauthmann et al., 2014): Duty (Does something need to be done?), Intellect (Is deep cognitive processing required?), Adversity (Is someone threatened by external forces?), Mating (Is there an opportunity to attract potential mates?), pOsitivity (Is the situation pleasant?), Negativity (Can the situation arouse negative feelings?), Deception (Can others be trusted?), and Sociality (Is social interaction possible or expected?). Research on undergraduate students by Sherman et al. (2013) revealed that individuals' personality and gender play a role in how individuals perceive daily life situations: Men estimated situations as holding more potential for blame, more potential for undermining or sabotage, and more potential for others to be “under threat.” Women were more likely to view situations with reference to their potential to evoke a need for support, to give rise to “warmth or compassion”, or to allow for emotional expression.

Taking into account the trend that contemporary approaches in the research on situation perception mainly focus on daily life situations (e.g., Sherman et al., 2010, 2013; Rauthmann, 2012; Rauthmann et al., 2015; Horstmann and Ziegler, 2018), psychologists have thus far learned little about the perception of high-stakes test situations. Bringing current findings on test bias (e.g., test anxiety) and contemporary research on situation perception together, this study aimed to shed light on a new viewpoint on testing focusing on the applicant's subjective perception of the situation as a previously unconsidered source of construct-irrelevant variance.

### The Present Study

**Figure 1:**
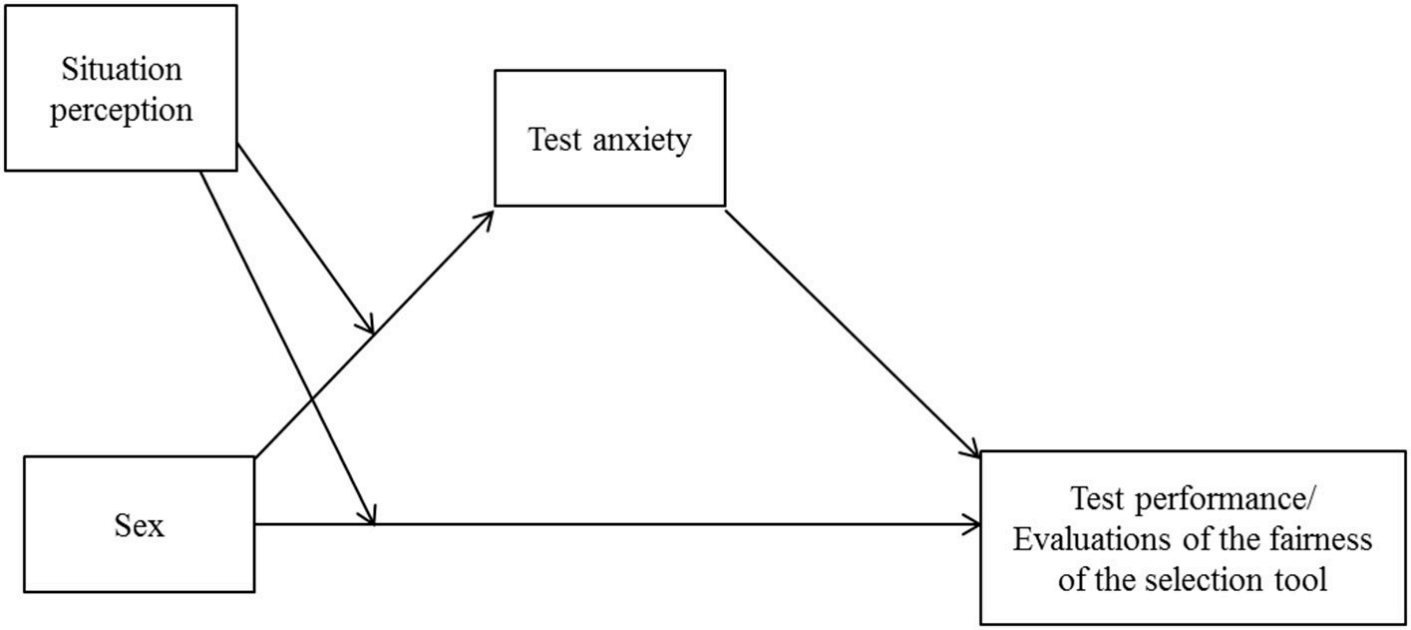
Conceptual diagram of the moderated mediation model.

In this study, we investigated situation perception in a high-stakes test situation and its relations to sex differences in test performance and fairness evaluations. We addressed situation perception and further included test anxiety (as a personality trait) as sources of systematic construct-irrelevant score variance. Test takers completed a short paper-pencil form after taking a medical school entrance examination. On the basis of previous research (Sherman et al., 2013), we expected sex differences in the perceived characteristics of the test situation (Hypothesis 1: There will be differences in women's and men's perceptions of a high-stakes test situation). Furthermore, we included test takers' test anxiety (see, e.g., Chapell et al., 2005) and analyzed its unique and moderated effect (by situation perception) on (1) overall test performance and (2) evaluations of the fairness of the selection tool. Given that a university entrance examination serves different interests, we considered possible outcomes of the test on the test taker's side as well as the institution's side: Whereas, test takers aim for admission, and past experiences may result in future expectations with reference to similar situations (see Helms, 2006), the perceived fairness of the testing tool is known to influence an organization's attractiveness (Chapman et al., 2005). We expected both variables, overall test performance as well as the evaluation of the fairness of the selection tool, to be influenced by test anxiety and therefore anticipated test anxiety to function as a suppressor variable in two ways: First, we expected general test anxiety to serve as a mediating variable between test takers' sex and test performance (Hypothesis 2: There will be an indirect effect of sex on performance through test anxiety, which will be moderated by the perception of the situation). Second, and in a similar manner, we expected that test anxiety would serve as a mediating variable between test takers' sex and their evaluation of whether the testing procedure was fair (Hypothesis 3: There will be an indirect effect of sex on evaluations of fairness through test anxiety, which will be moderated by the perception of the situation). Because the influence of situation perception has yet to be investigated in the context of high-stakes tests, we did not formulate directional predictions. However, we expected that aspects that reveal as relevant for situation perception in the context of high-stakes tests may serve as a possible moderating variable as presented in Figure 1.

## Methods

### Participants

In sum, 777 applicants took the entrance test at a private medical school in Austria. In a specially prepared lecture hall, every test taker was provided a workspace with a laptop and a computer mouse as well as a closed white envelope, which contained the evaluation form. After the test, the last screen informed the applicants that the test was over and invited them to open the envelope and voluntarily fill out the items. There were 25 test takers who did not return the evaluation form and five who answered < 50% of all items and were therefore not included in further analyses. The resulting sample consisted of 747 participants (442 women between the ages of 16 and 44, *M* = 20.64, *SD* = 2.66, and 305 men between the ages of 17 and 35, *M* = 21.10, *SD* = 2.56). The major group of participants was German citizens (60%), followed by 35% Austrian citizens, and the remaining 5% were citizens of other countries. The number of cases serving as a base for particular analyses was sometimes slightly smaller because some data were missing on specific scales.

### Procedure

The examination took place during 6 days in April 2017, with a maximum of two test sessions per day, one starting at 08:00 a.m. and one starting at 01:00 p.m. The computerized 4-h aptitude test consisted of 11 different subtests. After the test takers had completed the computerized aptitude battery, they were invited to fill out a short evaluation form. The evaluation form informed the test takers that the aim was to obtain test takers' evaluations of the test situation and test takers' experiences in order to enhance the test and the test situation in the future. The evaluation form included (1) items for assessing test takers' evaluations of the fairness of the testing tool, (2) items for assessing general test anxiety and situation perception as well as (3) an opportunity to provide feedback in a free-response format. Test takers were informed that the information they provided on the evaluation form would not have any impact on the admission decision and that there would not be a risk of harm due to their participation in the survey. Furthermore, test takers were informed that participation was voluntary and refusing had no consequences. On average, it took about 5 min to fill out the form.

### Materials

The evaluation form included short forms of existing scales for assessing the perceived fairness of the selection tool, test situation perception, and general test anxiety (see Table 1 for an overview of all items). It also included free-response evaluation items. Short scales of original questionnaires were administered to keep the form brief and to ensure that as many test takers as possible would fill it out voluntarily. Test takers rated each item on a 7-point rating scale (0 = *not at* all, 6 = *absolutely*), except one item concerning their overall evaluation of the fairness of the testing tool, to which they assigned a grade (A–E). To estimate the psychometric properties of the scales, we ran exploratory factor analyses (see section Statistical Analyses). The psychometric properties of the resulting scales are presented in **Table 4**.

**Table 1:** Overview of items included in the evaluation form.

**Measures**	**Scale**	
Situation perception	Duty	**Task-oriented thinking was required**.
		Participation was a necessity.
	Adversity	**I was put under pressure**.
		**The situation was uncomfortable**.
	Positivity	**The situation was interesting**.
		The situation was pleasant.
	Deception	**I could present myself as different from how I really am**.
		**It was possible to be dishonest with someone**.
	Sociality	**Communication with other people was important or desired**.
		**Close personal relationships were important or could develop**.
	Perceived strength: same construal	Procedures were precisely regulated and the same for everyone.
		It was possible to present oneself individually. (*reversed*)
	Perceived strength: appropriate response pattern	Every participant could behave as he/she thought best. (*reversed*)
	Perceived strength: adequate incentives	**There were personal incentives to complete the tasks as competently as possible**.
	Perceived strength: requirement of skills	One must possess certain skills in order to properly complete the tasks.
General test anxiety	Concern	**I worry about my performance**
	Lack of confidence	**I worry if I can make it at all.I am convinced that I will do well**. *(reversed)***I know that I can rely on myself**. (*reversed*)
Evaluation of the fairness of the selection tool	Measurement Quality	**The test makes it possible to measure performance differences between different people accurately**.
	Face Validity	**It is doubtful that this test can be used to identify qualified students**. (*reversed*)
	General Evaluation	**Which grade would you give the aptitude test you just took (A-E)?** (*reversed*)

Bold items were included in the analyses. Situation perception scales included original items from the S8* questionnaire (Rauthmann and Sherman, 2016) and adapted items, see Table 2.

#### Evaluation of the Fairness of the Selection Tool

We implemented a short version of the AKZEPT!-L survey (Kersting, 2008) in order to obtain test takers' subjective evaluations of the fairness of the selection tool. These comprised three items in total including the following aspects: *Measurement Quality, Face Validity* (both rated from 0 = *not at all* to 6 = *absolutely*), and an *Overall Evaluation* of the selection tool (graded from A-E; see Table 1).

#### Test Situation Perception

In order to obtain an individual score describing the subjective psychological quality of the test situation, we employed an adapted version of the S8^*^ questionnaire published by Rauthmann and Sherman (2016). The original S8^*^ questionnaire consists of 24 items (three items per each DIAMONDS dimension). For this study, we chose two items each from five of the eight original dimensions and adapted them to a test situation: *Duty, Adversity, pOsitivity, Deception*, and *Sociality*. A comparison of the original and adapted wording is presented in Table 2. This questionnaire had originally been developed for assessing perceptions in daily life situations.

**Table 2:** Original and adapted items from the S8^*^ questionnaire (Rauthmann and Sherman, 2016).

**Scale**	Original wording S8^*^	**Adapted wording for this study**
Duty	Task-oriented thinking is required.	Task-oriented thinking was required.
	I have to fulfill my duties.	Participation was a necessity.
Adversity	I am being threatened by someone or something.	I was put under pressure.
	I am being criticized.	The situation was uncomfortable.
Positivity	The situation is joyous and exuberant.	The situation was interesting.
	The situation is pleasant.	The situation was pleasant.
Deception	Not dealing with others in an honest way is possible.	I could present myself as different from how I really am.
	It is possible to deceive someone.	It was possible to be dishonest with someone.
Sociality	Communication with other people is important or desired.	Communication with other people was important or desired.
	Close personal relationships are important or could develop.	Close personal relationships were important or could develop.

*The adapted items were translated into English. The German originals can be obtained upon request*.

A high-stakes test situation differs from daily life situations, for example, in terms of its standardized structure and test taker's expected behavior, both of which are criteria for strong situations (Mischel, 1977). Therefore, we developed the items in accordance with Mischel's (1977) four criteria for strong situations, “leading everyone to construe the particular event in the same way,” “inducing uniform expectancies regarding the most appropriate response pattern,” “providing adequate incentives for the performance of the adequate response pattern,” and “requirement of skills that everyone has to the same extent” (rated from 0 = *not at all* to 6 = *absolutely*; see Table 1).

#### Test Anxiety

Test anxiety as a personality trait was assessed with four items from the short form of the Test Anxiety Inventory TAI-G—German version (TAI-G; Hodapp, 1991; rated from 0 = *not at all* to 6 = *absolutely*; see Table 1). Test takers were asked which statements were generally true for them when it comes to test situations. The original TAI-G questionnaire consists of 15 items (Wacker et al., 2008) and has been shown to assess more trait-related stable individual differences than situational effects (Keith et al., 2003).

#### Overall Test Performance on the Admission Test

Overall test performance was calculated as an average weighted z-standardized score of all subtests from the admission test for each test taker. This overall test score was comprised of results from 13 tests for assessing knowledge (e.g., basic knowledge in natural sciences, English), skills, or abilities (e.g., spatial ability, memory, reasoning). The overall score also included aspects of personality^1^ assessed with objective personality tests in computerized miniature situations (see Ortner and Proyer, 2015) and questionnaire items (see Ortner et al., 2017).

### Statistical Analyses

In order to estimate the psychometric properties of the adapted version of the S8^*^ questionnaire and Mischel's (1977) criteria for strong situations, we ran an exploratory factor analysis (principal axis factoring using oblimin rotation) to evaluate the factor structure. The results of parallel analysis as well as the scree plot suggested a four-factor solution. We therefore fixed the number of factors to four after dropping items due to low variance (one item) and low communality (<0.30; two items). Furthermore, we dropped items with factor loadings below 0.40 or substantial cross-loadings on several factors (three items). The final four-factor solution explained 64% of the variance and included nine items. The four resulting factors were labeled *Feeling stimulated, Opportunity to socialize, Feeling pressured*, and *Opportunity to deceive* (the items comprising each factor and the factor scores are presented in Table 3). The factor scores for these four factors were used for all further analyses (see the descriptive statistics and correlation coefficients for the resulting variables in Tables 4, 5).

**Table 3:** Factor loadings based on a principal components analysis with oblimin rotation for all items.

	**F1: Feeling pressured**	**F2: Opportunity to socialize**	**F3: Feeling stimulated**	**F4: Opportunity to deceive**
I was put under pressure	**0.86**	−0.04	0.15	0.05
The situation was uncomfortable	**0.47**	0.03	−0.28	0.06
Communication with other people was important or desired.	−0.02	**0.70**	0.01	−0.04
Close personal relationships were important or could develop.	0.01	**0.63**	0.01	0.04
The situation was interesting.	−0.07	0.03	**0.61**	0.16
There were personal incentives to complete the tasks as good as possible.	−0.03	−0.01	**0.42**	−0.04
Task-oriented thinking was required.	0.13	0.01	**0.40**	−0.15
It was possible to be dishonest with someone.	0.09	0.06	−0.13	**0.62**
I could present myself as different from how I really am.	0.01	0.00	0.06	**0.48**

*Final factor solution; factors were content-based and labeled as indicated in the table headings. Bold values indicate main factor loadings. N = 652*.

**Table 4:** Descriptive statistics for all dimensions.

	**#**	**α**	***N***	***M***	***SD***	**Min**	**Max**
1.Test anxiety	4	0.61	741	3.38	0.99	0.00	6.00
Situation perception							
2. Feeling stimulated	3	0.44	747	4.83	0.78	1.25	6.00
3. Opportunity to socialize	2	0.60	746	1.62	1.18	0.00	6.00
4. Feeling pressured	2	0.54	743	3.28	1.17	0.00	6.00
5. Opportunity to deceive	2	0.46	741	2.21	1.38	0.00	6.00
6. Evaluations of the fairness of the testing tool (z-score)	3	0.73	750	0.00	0.80	−2.36	1.88
7. Overall performance (z-score)	13	0.70	746	0.01	0.50	−1.97	1.80

*#, Number of items; α, Internal consistency*.

**Table 5:** Correlation coefficients (Pearson) between all scores.

	**Dimension**	**Subdimension**	**1**	**2**	**3**	**4**	**5**	**6**	**7**
1	Test anxiety		1.00						
2	Situation perception	Feeling stimulated	−0.17^**^	1.00					
3		Opportunity to socialize	−0.07	0.19^**^	1.00				
4		Feeling pressured	0.14^**^	−0.29^**^	−0.04	1.00			
5		Opportunity to deceive	−0.02	0.01	0.16^**^	0.15^**^	1.00		
6	Evaluations of the fairness of the testing tool (z)		−0.04	0.38^**^	−0.02	−0.22^**^	−0.18^**^	1.00	
7	Overall test performance (z)		−0.11^*^	0.06	−0.12^*^	0.02	0.05	0.14^**^	1.00

* p < 0.05,

** *p < 0.01*.

To address Hypothesis 1, whether men and women differ in their perceptions of a high-stakes test situation, we calculated simple *t*-tests with sex as the independent variable and four situation perception factors, which we obtained from the factor analysis, as the dependent variables.

We further analyzed whether the difference between men and women in the test results and in the evaluations of the fairness of the selection tool could be partly explained by different levels of general test anxiety (Hypotheses 2 and 3, respectively). Furthermore, we calculated whether this mediation would hold regardless of the extent to which test takers perceived the situation as a high-pressure situation during the test. For this purpose, we ran a mediation analysis in accordance with Hayes' guidelines (2013, Model 8) using PROCESS 2.16.3 for SPSS with sex as the independent variable, general test anxiety as the mediating variable, situation perception (*Feeling pressured*) as the moderating variable^2^, and the overall test result (Hypothesis 2) and fairness of the selection tool (Hypothesis 3) as the dependent variables (for an overview, see Figure 1). For all models, we centered the products of our variables and computed bias-corrected confidence intervals based on 5,000 bootstrapped samples.

## Results

An overview of the descriptive statistics for all scales is presented in Table 4. An overview of all correlations is presented in Table 5. Effect sizes are interpreted according to Cohen's (1988) classifications.

### Sex Differences in Test Situation Perception

For Hypothesis 1, addressing sex differences in the perception of the test situation, the analyses revealed significant albeit small differences in scores on the factor *Opportunity to deceive*, indicating slightly higher scores for men (*M* = 2.36, *SD* = 1.37) compared with women (*M* = 2.11, *SD* = 1.39), *t*_(739)_ = 2.45, *p* = 0.014, *d* = 0.18. This result indicates that men were slightly more likely to perceive the situation as an opportunity to engage in deception compared with women. The analyses further revealed differences with reference to the scores on the dimension *Opportunity to socialize*. The scores were higher for men (*M* = 1.26, *SD* = 1.40) than for women (*M* = 1.00, *SD* = 1.29), *t*_(726)_ = 2.62, *p* = 0.009, with a small effect size, *d* = 0.20. This result indicates that men reported viewing the test situation as more social than women did. Analyses showed a very small and non-significant difference in the situation perception dimension *Feeling pressured* [men: *M* = 3.31, *SD* = 1.30; women: *M* = 3.16, *SD* = 1.42, ΔM = 0.15, *t*_(741)_ = 1.50, *p* = 0.135, *d* = 0.11]. The analyses revealed no significant differences in situation perception with reference to the dimension *Feeling stimulated* (men: *M* = 4.87, *SD* = 0.86; women: *M* = 4.87, *SD* = 0.86), *t*_(745)_ = −0.43, *p* = 0.966, *d* = 0.00.

### Effects of Test Situation Perception and General Test Anxiety on Test Performance

**Figure 2:**
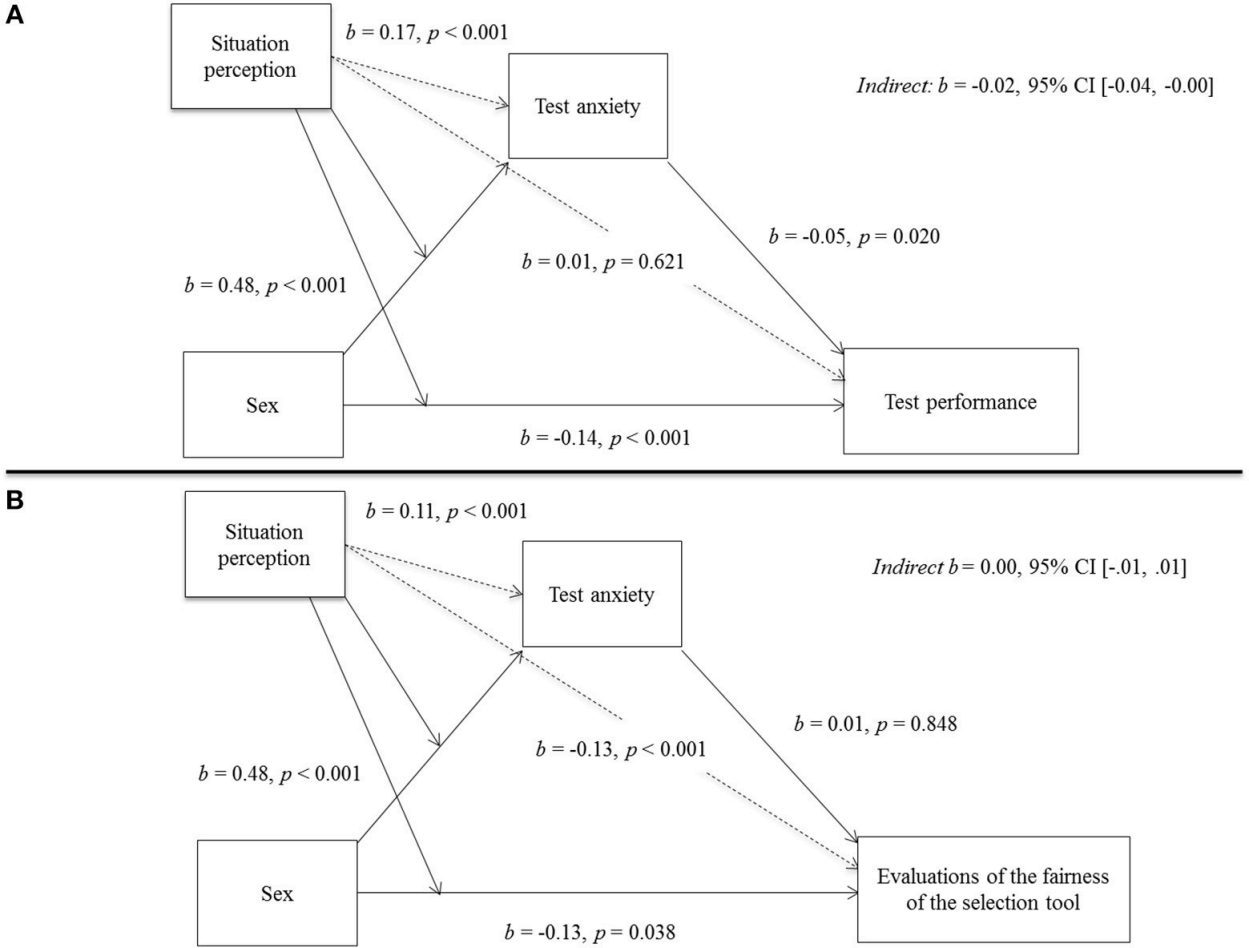
Test anxiety as mediator of the association between Sex and Overall performance **(A)** and Sex and Evaluations of the fairness of the selection tool **(B)**, moderated by Feeling pressured.

With reference to Hypothesis 2, we tested a moderated mediation model to assess the indirect effect of sex via test anxiety on overall test performance and to determine whether this indirect effect was influenced by the perception that the situation was a high-pressure situation (see also Figure 2A). In a first step, we reported the results (unstandardized regression coefficients including 95% bias-corrected bootstrapped confidence intervals with 5,000 samples) for the simple mediation model. Then we continued to check whether this indirect effect changed in accordance with test takers' perceptions of the situation (*Feeling pressured*). The results for the complete model are also presented in Table 6.

**Table 6:** Moderated mediation of sex predicting test anxiety and overall performance via feeling pressured.

**Predictor**		**Dependent variable**					
		**Test anxiety**			**Overall performance**		
		**b**	***SE***	***p***	**b**	***SE***	***p***
X	Sex	0.48	0.07	< 0.001	−0.14	0.04	< 0.001
M	Test anxiety	–	–	–	−0.05	0.02	0.020
W	Feeling pressured	0.11	0.03	< 0.001	0.01	0.01	0.471
X × W	Sex × Feeling pressured	−0.12	0.06	0.025	0.00	0.03	0.947
	Constant	3.38	0.04	< 0.001	0.16	0.07	0.019
		*R^2^* = 0.08			*R^2^* = 0.03		
		*F*_(3, 734)_ = 19.63, *p* < 0.001			*F*_(4, 733)_ = 6.79, *p* < 0.001		

*N = 730*.

The sex differences in overall test performance were significantly mediated by test anxiety (as indicated by a significant index of moderated mediation: *b* = 0.01, 95% CI [0.00; 0.02]). However, there was still a significant direct effect of sex on overall test performance (*b* = −0.15, *SE* = 0.04, *p* < 0.001), indicating that men scored higher than women on the test even after self-reported general test anxiety was entered as a mediator. Sex also had an effect on test anxiety: Women reported higher general test anxiety than men (*b* = 0.47, *SE* = 0.07, *p* < 0.001), and higher general test anxiety in turn led to lower overall performance (*b* = −0.04, *SE* = 0.02, *p* = 0.022).

**Figure 3:**
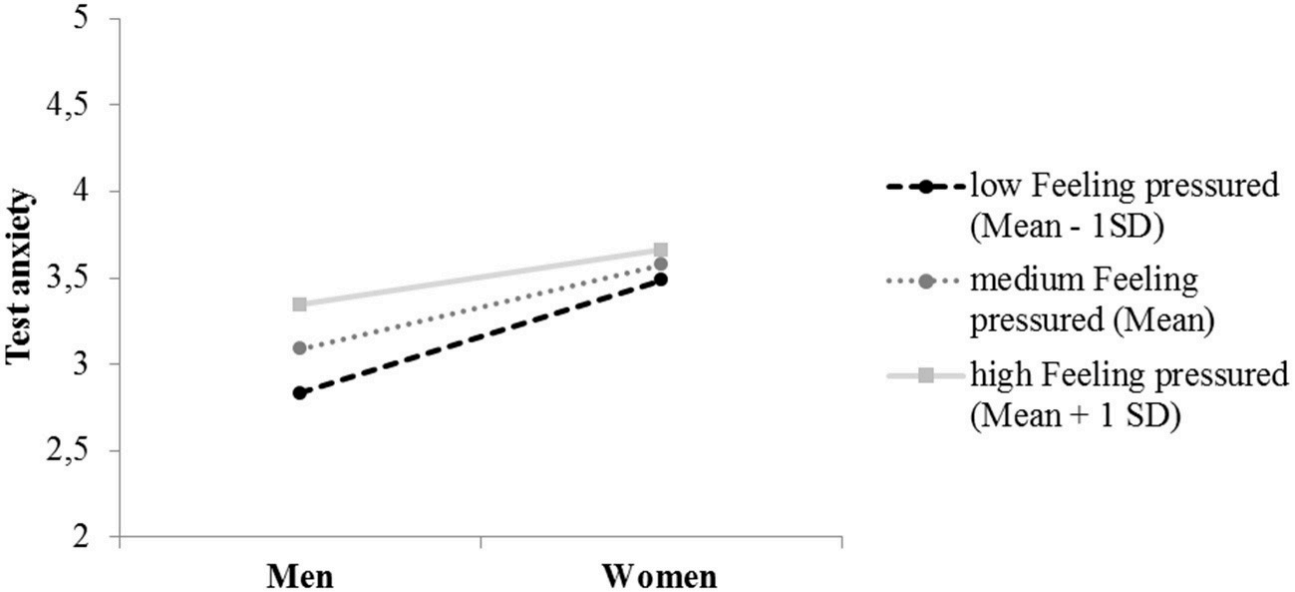
Test anxiety reported by women and men with reference to the different levels of *Feeling pressured*.

Differences in perceptions of the test situation concerning *Feeling pressured* did not affect performance (*b* = 0.08, *SE* = 0.02, *p* = 0.621) or the sex difference in performance (*b* = 0.01, *SE* = 0.03, *p* = 0.783). However, analyses revealed a significant positive relation between situation perception and test anxiety (*b* = 0.17, *SE* = 0.03, *p* < 0.001) and an effect of situation perception on the size of the sex difference in test anxiety (*b* = −0.18, *SE* = 0.07, *p* = 0.053): For people who reported low pressure (1 SD below the mean), the sex difference in test anxiety was higher (*b* = −0.03, *SE* = 0.01, 95% CI [−0.06, −0.01]) than for test takers who perceived the situation as a high-pressure situation (1 SD above the mean; *b* = −0.01, *SE* = 0.01, 95% CI [−0.03; −0.00]; see also Figure 3).

### Effects of test Situation Perception and General Test Anxiety on Fairness Evaluations

Parallel to the analyses used to address Hypothesis 2, we again tested a moderated mediation model to assess the extent of the indirect effect of sex via test anxiety on evaluations of fairness of the selection tool (Hypothesis 3). We also tested whether this indirect effect was influenced by the perception of the situation as a high-pressure situation (see Figure 2B). Again, we first reported the results for the simple mediation model. Then we continued to check whether this indirect effect changed in accordance with the perception of the situation.

The sex differences in evaluations of fairness of the selection tool were not significantly mediated by general test anxiety (indirect effect: *b* = −0.00, 95% CI [−0.01; 0.08]). However, there was still a significant direct effect of sex on evaluations of test fairness (*b* = −0.13, *SE* = 0.06, *p* = 0.038), indicating that men reported higher fairness ratings than women after self-reported test anxiety was entered as a mediator. Although sex had an effect on test anxiety, women reported higher test anxiety than men (*b* = 0.49, *SE* = 0.07, *p* < 0.001). Higher test anxiety in turn led to no change in the extent to which the selection tool was perceived to be fair (*b* = 0.01, *SE* = 0.03, *p* = 0.848).

Test takers' perceptions of the test situation concerning *Feeling pressured* did not affect the sex difference in the extent to which the selection tool was perceived to be fair (*b* = −0.03, *SE* = 0.07, *p* = 0.919) but had an effect on the size of the sex difference in test anxiety (*b* = −0.12, *SE* = 0.06, *p* = 0.025): The more the situation was perceived to be a high-pressure situation, the smaller the difference in self-reported test anxiety between men and women became. However, the perception of the situation as a high-pressure situation did not constitute a moderation of the indirect effect because the already mentioned effect of self-reported test anxiety on test takers' evaluation of the test was so low. Nevertheless, analyzes revealed an effect of *Feeling pressured* on the evaluations of the test situation: The more the situation was perceived to be a high-pressure situation, the lower the ratings of the test fairness were (*b* = −0.13, *SE* = 0.02, *p* < 0.001). The results for the model are also presented in Table 7 and in Figure 2B.

**Table 7:** Moderated mediation of sex predicting test anxiety and evaluations of the fairness of the selection tool via feeling pressured.

**Predictor**		**Dependent variable**					
		**Test anxiety**			**Evaluations of the fairness of the testing tool**		
		***b***	***SE***	***p***	***b***	***SE***	***p***
X	Sex	0.49	0.07	< 0.001	0.13	0.06	0.031
M	Test anxiety	–	–	–	−0.00	0.03	0.971
W	Feeling pressured	0.12	0.03	< 0.001	0.10	0.02	< 0.001
X × W	Sex × Feeling pressured	−0.18	0.06	0.037	0.00	0.03	0.947
Constant		3.38	0.04	< 0.001	2.62	0.12	< 0.001
		*R^2^ = 0.08*			*R^2^ = 0.03*		
		*F*_(3, 726)_ = 19.68, *p* < 0.001			*F*_(4, 725)_ = 4.99, *p =* 0.001		

*N = 730*.

## Discussion

This study was the first to investigate sex differences in the perception of a real high-stakes test situation and to address the question of whether observed differences between men and women in test performance and evaluations of the fairness of the test can be explained by taking into account a thus far disregarded source (i.e., situation perception) and a well-investigated source (i.e., test anxiety) of construct-irrelevant variance. To implement this new approach, we analyzed data from a real university aptitude test while also considering the test takers' evaluations of the test.

First, we hypothesized sex differences in test takers' perceptions of the test situation with respect to their perceptions of the characteristics of the situation, whether they felt pressured, whether they felt stimulated, and their perceived opportunities to socialize and to deceive. Analyses partly supported our expectations and revealed sex differences on the dimensions *Opportunity to deceive* and *Opportunity to socialize*: More than women, men seemed to view the test situation as an opportunity to be dishonest (“I could present myself as different from how I really am”; “It was possible to be dishonest with someone”) and as a situation that allowed social contact (“Communication with other people was important or desired”; “Close personal relationships were important or could develop”). This finding of higher scores for men with reference to deception is in line with research on differences in dishonest behavior in men and women (Ward and Beck, 1990) and with research on men's greater readiness to show social desirability in responding in personnel selection (Ones and Viswesvaran, 1998). However, the opportunities to cheat on this entrance examination were reduced to a minimum given the highly standardized test scenario, accompanied by several trained supervisors and the computerized test. The finding that men reported higher ratings with reference to social aspects goes against Sherman et al. (2013) results, which revealed higher scores for women on the social dimension. Thus, the different results may be explained by the different connotations of the items that were employed on the one hand, but they may also be a result of the different types of situations that were investigated: Whereas, Sherman et al. (2013) investigated situations in the context of daily life, we focused on an atypical situation: a high-stakes test situation. Taking the items into considerations in our study, it seems that men may have seen this high-stakes test more as an opportunity to interact, network, and compete against others (see e.g., Niederle and Vesterlund, 2007). However, the analyses did not reveal any sex differences on the dimensions *Feeling pressured* and *Feeling stimulated*: Men and women seemed to similarly perceive the test as a high-pressure situation and as stimulating. Evaluating the effect sizes of the sex differences, it appears that they are small, with a maximum of *d* = 0.20. However, it is important to notice that there was no kind of experimental manipulation, and test takers responded to a real situation (see Sherman et al., 2013; section Size of Effects)—a situation that was supposed to be the same for every person taking the test.

With respect to the second hypothesis, we expected an indirect effect of sex through test anxiety on overall test performance, influenced by the perception of the test situation as a high-pressure situation. Analyses revealed that men received higher overall test performance scores than women and that this finding could be attributed at least in part to an indirect negative causal effect of test anxiety. The lower overall test performance exhibited by women was partly explained by their higher general test anxiety, a finding that is in line with previous research (e.g., Osborne, 2001; Chapell et al., 2005) and indicates that construct-irrelevant variance was present to some extent in the test takers' results. Situation perception had no effect on overall test performance or on the connection between sex and test performance. However, there was a significant positive relation between the perception of the test situation as a high-pressure situation and general test anxiety: Higher scores of *Feeling pressured* were connected with higher test anxiety in men, which eventually led to the result that the difference in test anxiety between women and men was lowest in the group of test takers who particularly perceived the situation as a high-pressure situation (one standard deviation above the mean). This positive association between test anxiety and feelings of pressure supports Sherman et al. (2013) findings, which indicated that personality (in this case test anxiety) is a central and reliable component when it comes to differential situational construal.

Finally, with respect to the third hypothesis, we expected an indirect effect of sex on evaluations of fairness through test anxiety, influenced by the perception of the situation as a high-pressure situation. Results showed that women evaluated the selection tool as less fair in comparison with men. Whereas test anxiety did not affect the connection between test takers' sex and their fairness ratings, the data revealed that higher levels of *Feeling pressured* led to lower evaluations of the fairness of the selection tool. This negative relation indeed is not surprising because feeling pressured and inconvenienced during a test are important aspects of the overall evaluation of the test (e.g., Kersting, 2008). Therefore, the rather negative perceptions of the situation as high-pressure could in this sense also have reflected test takers' negative affective states, an interpretation that would be in line with Horstmann and Ziegler's (2018) results concerning the considerable overlap between the effects and perceptions of situations. However, negative attributions toward medical aptitude tests in Austria seem plausible, especially for women, given the annual reporting that casts doubt on the fairness of such proceedings (see e.g., online articles in kurier.at: Medizin-Aufnahmetest: Gender Gap bei Ergebnissen [Medical entrance test: Gender gap in results], 2015 and derstandard.at: Medizin-Aufnahmetest: Gender-Gap heuer wieder etwas größer [Medical entrance test: Gender gap this year slightly bigger again], 2017). Nevertheless, additional data with further independent measures of test situation construal and situational effects are needed in order to support or refute this argument.

When it comes to a competitive scenario, women face a different situation than men, as Gneezy et al. (2003) noted: “If women believe (even if incorrectly) that men are somewhat more skilled […] and they take the gender of their competitors as a signal of their ability (and maybe even take gender as a signal of their own ability), then a man and a woman face a different situation in the tournament” (p. 1058). These considerations are in line with gender roles, which classify women as highly qualified in communal scenarios and men as highly qualified in situations that call for assertiveness and mastery (Eagly and Miller, 2016). The results of several large studies (e.g., Colom et al., 2000; Colom and García-López, 2002; see also a review by Halpern and LaMay, 2000) have demonstrated that men and women are equivalent with reference to their general intelligence. However, men have been found to rate their own numerical IQ and their overall IQ higher than women do when it comes to self-estimated intelligence (Furnham et al., 2001; Ortner et al., 2011; see also a meta-analysis by Syzmanowicz and Furnham, 2011). Furnham et al. (2001) discussed the sex differences in self-estimations as influenced by lay conceptions about general intelligence and mathematical and spatial abilities, which are male normative. Such widely known stereotypes are supposed to impair the targets of these stereotypes, in this case women, and can be a driver of sex disparities when it comes to a high-stakes test situation.

Different reasons for the ongoing underrepresentation of women in STEM fields have been discussed (see e.g., Blickenstaff, 2005, for an overview), especially the effects of stereotype threat (Shapiro and Williams, 2012). Given the findings of this study, it seems reasonable to establish perceptions of the test situation as another approach in this context because test situations are an important part of a student's life, and they may have an important impact on career decisions. For example, research has revealed that higher grades in science, technology, engineering, and math (STEM) courses increase a student's probability of continuing with a STEM major (Griffith, 2010). Therefore, we advocate for more empirical research in this area to better understand the interplay between the situational characteristics of high-stakes situations, personality traits such as general test anxiety, and performance differences in men and women, especially in the light of consequences concerning further career implications.

## Limitations and Outlook

Several limitations need to be discussed in order to evaluate the given results. Due to the novelty of our research approach, the questions for assessing test takers' perceptions of the situation were based on an already existing form that was developed for investigations of daily life situations. We adapted the items, but we still think there is room for improvement in the formulation of the items so that they will better fit the special requirement of test-taking in a high-stakes situation. Future studies could thus seek to further develop this approach and, on the basis of the gained knowledge, use more selective items that can capture relevant aspects of the test situation.

The procedure of presenting the evaluation form directly after the 4-h aptitude test may have resulted in a too undifferentiated picture of test takers' perceptions because this procedure provided only an overall impression of the individuals' perceptions of the test situation. Nevertheless, it was not possible to evaluate the test situation in parts (e.g., after each task on the test) and to further examine whether the different tasks on the aptitude test induced different outcomes in test takers' perceptions. In addition, administering the evaluation form after the test might suffer from the disadvantage that test takers were fatigued, and asking participants about their perceptions of the test situation as well as their general test anxiety immediately after the admissions test may have led to ratings that were biased by expectations of success or frustration. Nevertheless, this limitation could not have been avoided because there was no opportunity to contact all of the participants before and after the admission procedure. In this regard, a reviewer raised the question of whether test anxiety may reflect a different type of anxiety that is related to the anticipated outcome of the high-stakes test. The assessment of test anxiety employed in this study (TAI-G; Wacker et al., 2008) was intended as an assessment of *general* test anxiety in order to avoid contaminations by a test taker's beliefs about his or her own performance. To make this purpose as clear as possible, participants were explicitly asked to respond to these items by stating what was generally true for them in test situations. Further, as referred to in section Test Anxiety, the TAI-G has been shown to assess more trait-related stable individual differences than situational effects (Keith et al., 2003). However, future research may include test takers' performance expectations as a covariate variable in order to avoid a possible impact of low performance expectations on the assessment of general test anxiety.

Finally, although the information the test takers provided was anonymous, and we made sure to emphasize that it would have no influence on the evaluations of the test takers' performance, we cannot be certain that the test takers' answers were free from social desirability. There have been discussions in the literature, for example, about the idea that even if men and women experience a condition similarly, women express their emotions differently (for an overview, see Vigil, 2009). Due to differences in gender roles, which prescribe appropriate behaviors for men and women (Eagly, 1987; Eagly and Wood, 1991), reporting negative cognitions such as anxiety may be less appropriate for men than for women (see e.g., Feingold, 1994). However, a qualitative analysis after a real-life testing scenario in which test takers are encouraged to answer the question of why a high-stakes test could generally, for women and men, be perceived as fear-triggering and unfair may be able to shed more light on this question. In this context, future research could further investigate the effect of the perceptions of a test situation on test performance in a controlled stereotype-free condition vs. a stereotype-threat condition. The perception of the test situation as positive and challenging, for example, could enhance women's motivation in a stereotype-free condition and serve as a buffer in a stereotype-threat condition.

## Conclusion

It is a practitioner's duty to provide every person who takes a test the same chance to show his or her knowledge, skills and abilities and to thereby follow the standards for test fairness (e.g., the Standards for Educational and Psychological Testing). However, the results of this study raise the question of the comparability of test situations for women and men. The present research contribution aimed to take a first step toward highlighting the importance of analyzing aspects of women's and men's different perceptions of an important test situation as a possible source of construct-irrelevant score variance, resulting in a contribution to sex differences in test performance that can have major impact on further career developments. Increasing knowledge of relevant influences may provide the chance to develop test situations or methods that minimize these effects and allow women to excel.

## Ethics Statement

This data collection was carried out in accordance with the recommendations of the American Psychological Association's Ethical Principles in the Conduct of Research with Human Participants. The protocol was approved by the Institutional Review Board at Salzburg University. All subjects filled out the evaluation form voluntarily and could withdraw at any time without any consequences.

## Author Contributions

All three authors developed the study concept. JL carried out the data collection and performed the data analysis under supervision of TS and TO. JL drafted an initial version of the manuscript that was refined and revisited successively by TO and TS.

### Conflict of Interest Statement

The authors declare that the research was conducted in the absence of any commercial or financial relationships that could be construed as a potential conflict of interest.

## Footnotes

^1^ Based on an empirical analysis of expert's evaluations, high conscientiousness as well as high agreeableness revealed as important requirements.

^2^ We exclusively report the *Feeling pressured* factor because other situation perception factors did not function as significant moderators of the postulated model. However, results of the moderated mediation models with the other three situation perception factors *Opportunity to socialize, Feeling stimulated* and *Opportunity to deceive* are available upon request.

## References

[B1] Aguinis H. Culpepper S. A. Pierce C. A. (2016). Differential prediction generalization in college admissions testing. J. Educ. Psychol. 108, 1045–1059. 10.1037/edu0000104

[B2] American Educational Research Association, American Psychological, Association, and National Council on Measurement in Education. (2014). Standards for Educational and Psychological Testing. Washington, DC: American Educational Research Association.

[B3] Ashcraft M. H. Kirk E. P. (2001). The relationships among working memory, math anxiety, and performance. J. Exp. Psychol. 130, 224–237. 10.1037/0096-3445.130.2.224 11409101

[B4] Backus P. Cubel M. Guid M. Sánchez-Pages S. Manas E. L. (2016). Gender, Competition and Performance: Evidence From Real Tournaments. Working Paper 2016-27, Institut d'Economia da Barcelona (IEB). 10.2139/ssrn.2858984

[B5] Blickenstaff J. C. (2005). Women and science careers: leaky pipeline or gender filter? Gender Educ. 17, 369–386. 10.1080/09540250500145072

[B6] Bonaccio S. Reeve C. L. (2010). The nature and relative importance of students' perceptions of the sources of test anxiety. Learn. Individ. Differ. 20, 617–625. 10.1016/j.lindif.2010.09.007

[B7] Cadinu M. Maass A. Rosabianca A. Kiesner J. (2005). Why do women underperform under stereotype threat? Evidence for the role of negative thinking. Psychol. Sci. 16, 572–578. 10.1111/j.0956-7976.2005.01577.x 16008792

[B8] Cassady J. C. Johnson R. E. (2002). Cognitive test anxiety and academic performance. Contemp. Educ. Psychol. 27, 270–295. 10.1006/ceps.2001.1094

[B9] Chapell M. S. Blanding Z. B. Silverstein M. E. Takahashi M. Newman B. Gubi A. (2005). Test anxiety and academic performance in undergraduate and graduate students. J. Educ. Psychol. 97, 268–274. 10.1037/0022-0663.97.2.268

[B10] Chapman D. S. Uggerslev K. L. Carroll S. A. Piasentin K. A. Jones D. A. (2005). Applicant attraction to organizations and job choice: a meta-analytic review of the correlates of recruiting outcomes. J. Appl. Psychol. 90, 928–944. 10.1037/0021-9010.90.5.928 16162065

[B11] Cohen J. (1988). Statistical Power Analysis for the Behavioral Sciences, 2nd Edn. Hillsdale, NJ: Erlbaum.

[B12] Colom R. García-López O. (2002). Sex differences in fluid intelligence among high school graduates. Pers. Individ. Dif. 32, 445–451. 10.1016/S0191-8869(01)00040-X

[B13] Colom R. Juan-Espinosa M. Abad F. García L. F. (2000). Negligible sex differences in general intelligence. Intelligence 28, 57–68. 10.1016/S0160-2896(99)00035-5 12025362

[B14] Cooper W. H. Withey M. J. (2009). The strong situation hypothesis. Pers. Soc. Psychol. Rev. 13, 62–72. 10.1177/1088868308329378 19144905

[B15] Cote J. A. Buckley R. (1987). Estimating trait, method, and error variance: generalizing across 70 construct validation studies. J. Market. Res. 24, 315–318. 10.2307/3151642

[B16] Eagly A. H. (1987). Reporting sex differences. Am. Psychol. 42, 756–757. 10.1037/0003-066X.42.7.755

[B17] Eagly A. H. Miller D. I. (2016). Scientific eminence: where are the women? Perspect. Psychol. Sci. 11, 899–904. 10.1177/1745691616663918 27899733

[B18] Eagly A. H. Wood W. (1991). Explaining sex differences in social behavior: a meta-analytic perspective. Pers. Soc. Psychol. Bull. 17, 306–315. 10.1177/0146167291173011

[B19] Edwards J. Templeton A. (2005). The structure of perceived qualities of situations. Eur. J. Soc. Psychol. 35, 705–723. 10.1002/ejsp.271

[B20] Else-Quest N. M. Hyde J. S. Linn M. C. (2010). Cross-national patterns of gender differences in mathematics: a meta-analysis. Psychol. Bull. 136, 103–127. 10.1037/a0018053 20063928

[B21] EU (2012). She Figures. Gender in Research and Innovation. Brussels: European Commission.

[B22] Eysenck M. W. Calvo M. G. (1992). Anxiety and performance: the processing efficiency theory. Cogn. Emot. 6, 409–434. 10.1080/02699939208409696

[B23] Feingold A. (1994). Gender differences in personality: a meta-analysis. Psychol. Bull. 116, 429–456. 10.1037/0033-2909.116.3.429 7809307

[B24] Fischer F. T. Schult J. Hell B. (2013). Sex-specific differential prediction of college admission tests: a meta-analysis. J. Educ. Psychol. 105, 478–488. 10.1037/a0031956

[B25] Furnham A. Hosoe T. Tang T. L.-P. (2001). Male hubris and female humility? A crosscultural study of ratings of self, parental, and sibling multiple intelligence in America, Britain, and Japan. Intelligence 30, 101–115. 10.1016/S0160-2896(01)00080-0

[B26] Gneezy U. Niederle M. Rustichini A. (2003). Performance in competitive environments: gender differences. Q. J. Econ. 118, 1049–1074. 10.1162/00335530360698496

[B27] Griffith A. L. (2010). Persistence of Women and Minorities in STEM Field Majors: Is it the School That Matters? Cornwell University; School of Industrial and Labor. Available online at: https://digitalcommons.ilr.cornell.edu/workingpapers/122/ (Accessed July 19, 2018).

[B28] Haladyna T. M. Downing S. M. (2004). Construct-irrelevant variance in high-stakes testing. Educ. Meas. 23, 17–27. 10.1111/j.1745-3992.2004.tb00149.x

[B29] Halpern D. F. LaMay M. L. (2000). The smarter sex: a critical review of sex differences in intelligence. Educ. Psychol. Rev. 12, 229–246. 10.1023/A:1009027516424

[B30] Hancock D. R. (2001). Effects of test anxiety and evaluative threat on student's achievement and motivation. J. Educ. Res. 94, 284–290. 10.1080/00220670109598764

[B31] Helms J. E. (2006). Fairness is not validity or cultural bias in racial-group assessment: a quantitative perspective. Am. Psychol. 61, 845–859. 10.1037/0003-066X.61.8.845 17115831

[B32] Hembree R. (1988). Correlates, causes, effects, and treatment of test anxiety. Rev. Educ. Res. 58, 47–77. 10.3102/00346543058001047

[B33] Hodapp V. (1991). Das Prüfungsängstlichkeitsinventar TAI-G: eine erweiterte und modifizierte Version mit vier Komponenten [The Test Anxiety Inventory TAI-G: an expanded and modified version with four components]. Z. Pädagogische Psychol. 5, 121–130.

[B34] Horstmann K. T. Ziegler M. (2018). Situational perception and affect: barking up the wrong tree? Pers. Individ. Differ. 136, 132–139. 10.1016/j.paid.2018.01.020

[B35] Hyde J. S. Fennema E. Lamon S. J. (1990). Gender differences in mathematics performance: a meta-analysis. Psychol. Bull. 107, 139–155. 10.1037/0033-2909.107.2.139 2138794

[B36] Kaufmann J. C. (2010). Using creativity to reduce ethnic bias in college admissions. Rev. Gen. Psychol. 14, 189–203. 10.1037/a0020133

[B37] Keith N. Hodapp V. Schernelleh-engel K. Moosbrugger H. (2003). Cross-sectional and longitudinal confirmatory factor models for the German test anxiety inventory: a construct validation. Anxiety Stress Cop. 16, 251–270. 10.1080/1061580031000095416

[B38] Kersting M. (2008). Zur Akzeptanz von Intelligenz- und Leistungstests. Towards the fairness evaluation of intelligence and performance tests. Rep. Psychol. 33, 420–430.

[B39] Konegen-Grenier C. (2018). Wer Bekommt Einen Studienplatz? Die Regelung des Hochschulzugangs im Umbruch [Who Gets a Place to Study? The Regulation of University Entrance in Transition]. IW-Report, No. 22/2018, Institut der deutschen Wirtschaft Köln (IW), Köln. Avaialble online at: https://www.econstor.eu/bitstream/10419/180024/1/1025146530.pdf

[B40] Lewin K. (1946). Behavior and development as a function of the total situation, in Manual of Child Psychology, ed Carmichael L. (Hoboken, NJ: John Wiley & Sons Inc), 791–844.

[B41] Mau W.-C. Lynn R. (2001). Gender differences on the scholastic aptitude test, the American college test and college grades. Educ. Psychol. 21, 133–136. 10.1080/01443410020043832

[B42] Medizin-Aufnahmetest: Gender Gap bei Ergebnissen [Medical entrance test: Gender gap in results] (2015). Available online at: Retreived from https://kurier.at/politik/inland/medizin-aufnahmetest-geschlechtsunterschiede-bei-ergebnissen/145.522.410 (Accesses September 03, 2018).

[B43] Medizin-Aufnahmetest: Gender-Gap heuer wieder etwas größer [Medical entrance test: Gender gap this year slightly bigger again] (2017). www.derstandard.at. Available online at: https://derstandard.at/2000062402011/Medizin-Aufnahmetest-Gender-Gap-heuer-wieder-etwas-groesser (Accesses September 03, 2018).

[B44] Mischel W. (1977). The interaction of person and situation, in Personality at the Cross-Rods: Current Issues in Interactional Psychology, eds Magnusson D. Endler N. S (Hillsdale, NJ: Lawrence Erlbaum), 333–352.

[B45] Niederle M. Vesterlund L. (2007). Do women shy away from competition? Do men compete too much? Q. J. Econ. 122, 1067–1101. 10.1162/qjec.122.3.1067

[B46] Niederle M. Vesterlund L. (2010). Explaining the gender gap in math test scores: the role of competition. J. Econ. Perspect. 24, 129–144. 10.1257/jep.24.2.129

[B47] Ones D. S. Viswesvaran C. (1998). The effects of social desirability and faking on personality and integrity assessment for personnel selection. Hum. Perform. 11, 245–269. 10.1080/08959285.1998.9668033

[B48] Ortner T. M. Augart S. Leiner J. Scherndl T. (2017). Ein neuer Eignungstest für das Medizinstudium an der PMU: a new test for the assessment of aptitude for the study of medicine, in Poster presented at the Gesellschaft für Medizinische Ausbildung (GMA) (Vienna).

[B49] Ortner T. M. Müller S. M. Garcia-Retamero R. (2011). Estimations of parental and self intelligence as a function of parents' status: a cross-cultural study in Germany and Spain. Soc. Sci. Res. 40, 1067–1077. 10.1016/j.ssresearch.2011.03.006

[B50] Ortner T. M. Proyer R. T. (2015). Objective personality tests, in Behavior Based Assessment in Psychology, eds van de Vijver F. J. Ortner T. M (Göttingen: Hogrefe), 133–149.

[B51] Osborne J. W. (2001). Testing stereotype threat: does anxiety explain race and sex differences in achievement? Contemp. Educ. Psychol. 26, 291–310. 10.1006/ceps.2000.1052 11414722

[B52] Pfarrhofer H. (2017). Medizin-Aufnahmetest: Männer Sind Erfolgreicher als Frauen ?Admission test for Medicine: Men Are More Successful Than Women. DiePresse.com. Available online at: https://diepresse.com/home/bildung/universitaet/5265904/MedizinAufnahmetest_Maenner-sind-erfolgreicher-als-Frauen (Accessed January 31, 2018).

[B53] Rauthmann J. F. (2012). You say the party is dull, I say it is lively: a componential approach to how situations are perceived to disentangle perceiver, situation, and perceiver x situation variance. Soc. Psychol. Pers. Sci. 3, 519–528. 10.1177/1948550611427609

[B54] Rauthmann J. F. Gallardo-Pujol D. Guillaume E. M. Todd E. Nave C. S. Sherman R. A.. (2014). The situational eight DIAMONDS: a taxonomy of major dimensions of situation characteristics. J. Pers. Soc. Psychol. 107, 677–718. 10.1037/a0037250 25133715

[B55] Rauthmann J. F. Horstmann K. T. Sherman R. A. (2018). Do self-reported traits and aggregated states capture the same thing? A nomological perspective on trait-state homomorphy. Soc. Psychol. Pers. Sci. 10.1177/1948550618774772

[B56] Rauthmann J. F. Sherman R. A. (2016). Measuring the Situational Eight DIAMONDS characteristics of situations: an optimization of the RSQ-8 to the S8^*^. Eur. J. Psychol. Assess. 32, 155–164. 10.1027/1015-5759/a000246

[B57] Rauthmann J. F. Sherman R. A. (2017). Normative and distinctive accuracy in situation perceptions. Soc. Psychol. Pers. Sci. 8, 768–779. 10.1177/1948550616687095

[B58] Rauthmann J. F. Sherman R. A. Nave C. S. Funder D. C. (2015). Personality-driven situation experience, contact, and construal: how people's personality traits predict characteristics of their situations in daily life. J. Res. Pers. 55, 98–111. 10.1016/j.jrp.2015.02.003

[B59] Reilly D. Neumann D. L. Andrews G. (2015). Sex differences in mathematics and science achievement: a meta-analysis of national assessment of educational progress assessments. J. Educ. Psychol. 107, 645–662. 10.1037/edu0000012

[B60] Reilly D. Neumann D. L. Andrews G. (2018). Gender differences in reading and writing achievement: evidence from the national assessment of educational progress (NAEP). Am. Psychol. [Epub ahead of print]. 10.1037/amp0000356 30234314

[B61] Salchegger S. Suchan B. (2018). Was bedeutet es für den Geschlechterunterschied in der Mathematikkompetenz bei PISA, wenn dem Schulsystem leistungsschwache Jungen verloren gehen? [What does it mean for the gender difference in mathematics literacy in PISA if the school system loses under-performing boys?]. Z. Bildungsforsch. 8, 81–99. 10.1007/s35834-017-0190-7

[B62] Sarason I. G. (1978). The test anxiety scale: concept and research, in Stress and Anxiety: Vol. 5, eds Spielberger C. D. Sarason I. G (Washington, DC: Hemisphere Publishing Corporation), 193–216.

[B63] Sarason I. G. Sarason B. R. (1990). Test anxiety, in Handbook of Social and Evaluative Anxiety, ed Leitenberg H (New York, NY: Plenum Press), 475–496. 10.1007/978-1-4899-2504-6_16

[B64] Schmader T. Johns M. (2003). Converging evidence that stereotype threat reduces working memory capacity. J. Pers. Soc. Psychol. 85. 440–452. 10.1037/0022-3514.85.3.440 14498781

[B65] Shapiro J. R. Williams A. M. (2012). The role of stereotype threats in undermining girls' and women's performance and interest in STEM fields. Sex Roles 66, 175–183. 10.1007/s11199-011-0051-0

[B66] Sherman R. A. Nave C. S. Funder D. C. (2010). Situational similarity and personality predict behavioral consistency. J. Pers. Soc. Psychol. 99, 330–343. 10.1037/a0019796 20658847

[B67] Sherman R. A. Nave C. S. Funder D. C. (2013). Situational construal is related to personality and gender. J. Res. Pers. 47, 1–14. 10.1016/j.jrp.2012.10.008

[B68] Spencer S. J. Steele C. M. Quinn D. M. (1999). Stereotype threat and women's math performance. J. Exp. Soc. Psychol. 35, 4–28. 10.1006/jesp.1998.1373

[B69] Steele C. M. (1997). A threat in the air: how stereotypes shape intellectual identity and performance. Am. Psychol. 52, 613–629. 10.1037/0003-066X.52.6.613 9174398

[B70] Stone E. A. Cook L. L. (2016). Testing individuals in special populations, in Fairness in Educational Assessment and Measurement, eds Dorans N. J. Cook L. L (New York, NY: Routledge), 157–180.

[B71] Syzmanowicz A. Furnham A. (2011). Gender differences in self-estimates of general, mathematical, spatial and verbal intelligence: four meta analyses. Learn. Individ. Differ. 21, 493–504. 10.1016/j.lindif.2011.07.001

[B72] Turner C. Mintz L. Carr F. (2017). Rise in Top Universities Setting Own Entrance Exams as They Cannot Rely on A-Levels. www.telegraph.co.uk. Available online at: https://www.telegraph.co.uk/education/2017/08/19/rise-top-universities-setting-entrance-exams-cannot-rely-a-levels/ (Accessed May 22, 2018).

[B73] Vigil J. M. (2009). A socio-relational framework of sex differences in the expression of emotion. Behav. Brain Sci. 32, 375–428. 10.1017/S0140525X09991075 19825246

[B74] Wacker A. Jaunzeme J. Jaksztat S. (2008). Eine Kurzform des Prüfungsängstlichkeitsinventars TAI-G. Z. Pädagogische Psychol. 22, 73–81. 10.1024/1010-0652.22.1.73

[B75] Wagerman S. Funder D. (2009). Personality psychology of situations, in The Cambridge Handbook of Personality Psychology, eds Corr P. J. Matthews G (New York, NY: Cambridge University Press), 27–42.

[B76] Ward D. A. Beck W. L. (1990). Gender and dishonesty. J. Soc. Psychol. 130, 333–339. 10.1080/00224545.1990.9924589

[B77] Willingham W. W. Cole N. S. (1997). Test performance, in Gender and Fair Assessment, eds Willingham W. W. Cole N. S (Mahwah, NJ: Erlbaum), 55–126.

[B78] Zeidner M. (1990). Does test anxiety bias scholastic aptitude test performance by gender and sociocultural group? J. Pers. Assess. 55, 145–160. 2231237 10.1080/00223891.1990.9674054

[B79] Zeidner M. Schleyer E. J. (1998). The big-fish–little-pond effect for academic self-concept, test anxiety, and school grades in gifted children. Contemp. Educ. Psychol. 24, 305–329. 10.1006/ceps.1998.0985 10508530

